# Restorative Just Culture: An Exploration of the Enabling Conditions for Successful Implementation

**DOI:** 10.3390/healthcare12202046

**Published:** 2024-10-15

**Authors:** Leonie Boskeljon-Horst, Vincent Steinmetz, Sidney Dekker

**Affiliations:** 1 Netherlands Defence Academy, Isaac Delprat Paviljoen, Hogeschoollaan 2, 4818 BB Breda, The Netherlands; 2 Voqx—Innovative Safety, Willem van Oranjelaan 21, 1412 GJ Naarden, The Netherlands; v.steinmetz@voqx.nl; 3 Safety Science Innovation Lab, School of Humanities, Languages and Social Science, Griffith University, 170 Kessels Road, Nathan Campus, Nathan, QLD 4111, Australia; s.dekker@griffith.edu.au

**Keywords:** restorative just culture, enabling conditions, leadership qualities, second victim engagement

## Abstract

Background/Objectives: Restorative responses to staff involved in incidents are becoming recognized as a rigorous and constructive alternative to retributive forms of ‘just culture’. However, actually achieving restoration in mostly retributive working environments can be quite difficult. The conditions for the fair and successful application of restorative practices have not yet been established. In this article, we explore possible commonalities in the conditions for success across multiple cases and industries. Methods: In an exploratory review we analysed published and unpublished cases to discover enabling conditions. Results: We found eight enabling conditions—leadership response, leadership expectations, perspective of leadership, ‘tough on content, soft on relationships’, public and media attention, regulatory or judicial attention to the incident, second victim acknowledgement, and possible full-disclosure setting—whose absence or presence either hampered or fostered a restorative response. Conclusions: The enabling conditions seemed to coagulate around leadership qualities, media and judicial attention resulting in leadership apprehension or unease linked to their political room for maneuver in the wake of an incident, and the engagement of the ‘second victim’. These three categories can possibly form a frame within which the application of restorative justice can have a sustainable effect. Follow-up research is needed to test this hypothesis.

## 1. Introduction

Restorative justice is becoming recognized as an important component in the ongoing quest for improving patient safety and healthcare quality. A number of converging trends and developments are responsible for this. They include the growing acknowledgment of healthcare providers as second victims of clinical errors and incidents [1,2,3], which can lead to moral injury [4] and worse [5]; ethical innovations in our thinking about medical error and accountability [6,7]; data showing that honesty and disclosure are cheaper than denial and lawsuits [8]; and the realization that we cannot punish and threaten our way toward a safer healthcare system [9,10,11,12] but should be creating work climates of trust and openness [13,14,15,16]. Some of these insights have come on the back of highly public inquiries into the quality failings of healthcare [17].

Restorative responses to healthcare staff involved in incidents are becoming recognized as a rigorous and constructive alternative to retributive forms of ‘just culture’. The latter have typically been offered to healthcare leaders in the form of algorithms for establishing putative culpability and typically result in warnings, suspensions, and similar sanctions (e.g., [18,19]). The effects of ‘being just cultured’ on staff and the organization can be deleterious. Documented outcomes in studies in healthcare and other fields show that retributive responses can undermine the mental health, engagement, and motivation of those involved [20,21]; violate the rights and exacerbate the suffering of ‘second victims’ [1,2,3,22,23,24,25]; impede or even harm organizational learning and performance [8,26,27,28]; increase defensive posturing and safety clutter [29]; worsen staff insularity and silence [30,31]; exacerbate inequalities across medical-competency hierarchies in healthcare organizations; and invite abuses of power [32]. Restorative practices, in contrast, have yielded demonstrable benefits for second-victim experiences in healthcare [33] for claims resolution, stakeholder inclusion, quality of recommendations, just outcomes, risk awareness, and organizational learning [6,34,35,36,37].

Restorative just culture doesn’t ask so much about rules, violations, and proportional consequences but instead inquires after the impacts that an incident has had on people; the needs that this has created within and around them; and the obligations on stakeholders of all kinds that this produces [26]. Those involved may engage in processes and rituals for confession (disclosure), repentance (expressing remorse), and atonement (making things right), which can lead to (secular forms of) forgiveness, the redress of harm, and the repair of relationships of trust needed to continue working with or for each other [6]. The aspiration of accountability in this is forward-looking, attached to people’s roles and responsibilities, and oriented toward setting each other up for success going forward [7,38].

Conditions for the ‘fair’ application of retributive mechanisms—the principles of natural justice—such as an independent judge, a ‘jury’ of peers, and ways to appeal the outcome, have long been established [25,39], if variably followed inside healthcare and other organizations [20]. But similar meta-analyses for the fair and successful application of restorative practices in healthcare organizations have lagged behind the reporting of individual implementation initiatives [36]. Recent case research shows the application of restorative just culture, which failed to generate significant or sustainable success, even though appropriate steps had been followed [40]. In this instance, a spontaneous restorative response was observed, but an actual restorative outcome was only partially attained, in part because of a predominantly retributive organizational setting. It was concluded that a perceived tension between punishment and learning, the dilemma of choosing between one’s own career and applying a restorative response, and the price of vulnerability were among the factors hindering a sustainable restorative just culture [40].

A few applications of restorative practices within healthcare have been documented: for instance, the introduction of a Just Culture Guide by NHS Improvement. Although meant with good intentions, the focus in the Just Culture Guide on individual failing made it more of a retributive than a restorative measure [41]. Stretton [42] concludes that survey results as well as individual cases show that changes made to achieve a just culture are rather superficial and entail more symbolic than actual improvements. Based on an integrative review, Barkell and Stockton-Snyder [43] concluded that the empirical evidence regarding the application and benefits of just culture in healthcare is scarce.

On the other hand, Marrée [44] found that a restorative just culture increased safety, improved the relationship between patient and caregiver, and increased satisfaction with the way incidents are handled. Flores [45] evaluated two restorative just culture interventions using community-building circles. This evaluation showed that the interventions positively impacted interpersonal relationships; changed perspectives; fostered diverse, equitable and inclusive learning environments; addressed climate concerns such as bullying within the organization; and increased psychological safety.

According to Marréé [44], the literature points to five necessary, albeit general, conditions to successfully implement a restorative just culture, which are restorative just culture training, peer support networks, learning after an incident, disclosure, and inclusivity for all stakeholders. Kooijman [46] concluded that the Hippocratic oath, an institutional learning process after incidents, and building a community, i.e., strengthening the relationships between people, can be seen as facilitators for a restorative approach. Based on a systematic review, Murray et al. [47] found four (again, rather generic) themes that provide insights into the requirements for implementing a just culture in healthcare: leadership commitment, education and training, accountability, and open communication. They also conclude, however, that most published literature regarding just culture is theoretical and that more research into requirements is necessary in order to reach a sustainable restorative just culture.

After conducting a scoping review, Sawin et al. [48] found barriers that hamper the implementation of a restorative just culture, which included historical hierarchies in an organization, the required investment of time and resources into restorative justice, resistance and opposition to restorative approaches, and longstanding traditions that favor a retributive approach. Takser, Jones, and Brake [49] identified unfamiliarity with the concept of just culture, the management of incidents and incident reports, and a lack of formal training for investigators as barriers to achieving a just culture.

Wailling et al. [50] concluded, however, that although benefits of a restorative approach can be found, the same cannot be said for the identification of specific preconditions for a restorative just culture, which is required to achieve a successful implementation. Notwithstanding the few cases reported, the literature on the application of restorative just culture paradigms in healthcare is sufficiently sparse to encourage further exploration of possible commonalities in the conditions for success across multiple cases. Although a positive change in the just culture has been observed within healthcare organizations, the conditions necessary to not only start such a change but make it sustainable remain unclear [36]. In this paper, we conduct the first empirical exploration of such enabling conditions—the kinds of conditions or capacities that need to be in place, or constraints that need to be managed effectively, to form a frame within which the application of restorative justice can be successful.

## 2. Method

For the current paper, we used an exploratory approach combining published cases of restorative or retributive responses to incidents in healthcare as well as other fields [20,34,35,36,37,51,52]. We needed to be fairly open-minded about which fields of practice to include, given the tiny number of documented cases in healthcare alone. Given that these published cases do not explicitly talk about ‘enabling conditions’, and that all took place within one field or setting without the possibility of formal or systematic triangulation with other empirical material, we elected to test our initial gleanings from these published sources against our experiences with four unpublished (operational) cases. More specifically, we reviewed published cases in the literature to gain an idea, followed by a thorough review of published cases that we were involved in as researchers, and compared these to the four unpublished cases, of which we have in-depth knowledge as a result of our involvement. As part of this exploratory approach, we distilled enabling and hampering conditions regarding a restorative just culture and elicited general themes from these that seem to provide enabling conditions for successfully implementing a restorative just culture.

Our review consisted of several rounds. After getting a basic idea from the literature, the first level of analysis consisted of the open coding of the transcripts of our published and unpublished cases. Relevant findings were marked. In the second level of analysis, comparison of the relevant findings resulted in underlying themes. Finally, these themes resulted in the enabling conditions that seem to be needed for the successful implementation of a restorative just culture.

The details of the three published and four unpublished cases are as follows: the three published cases, of which we have in-depth knowledge, come from the Dutch defense organization, as do two of the four unpublished cases. One of the unpublished cases is a case from a healthcare organization (hospital) in the Netherlands, and the last of the unpublished cases comes from a European airline.

Two of the authors have been an observer or facilitator in the aftermath of the incidents described (LBH: the three published cases and unpublished cases 2 and 3; VS: unpublished case 1 and 4).

Published case 1 contains a case study based on in-depth individual and focus group interviews, as well as observations. Published case 2 contains a case study based on in-depth individual and focus group interviews, as well as observations. Published case 3 contains a case study based on in-depth individual interviews. Unpublished case 1 contains a case study in which the author was involved in the initiation of the restorative just culture process as an alternative to the initially proposed complaint process. Unpublished case 2 contains a case study based on an interview with an involved operator. Unpublished case 3 contains a case study based on a restorative meeting with the involved parties. Unpublished case 4 contains a case study detailing a process (flight replay) developed by the operator, its safety department, and the pilots’ union. The author (VS) chaired numerous replays.

These seven published and unpublished cases all contained and lacked elements which, prima facie, would have constituted important enabling conditions for restorative justice to succeed. In lieu of experimental design, the make-up of the additional unpublished cases allowed us to begin to test and control for possible confounds in the reported studies.

The published cases were as follows:

Case 1: Safety standdown: A pilot made judgement calls that made his CO doubt his capabilities. The CO sat down with him to hear his side of the story. He was satisfied with the pilot’s explanations, remorse, and sense of responsibility, and the pilot received no official warning. In the months following, the pilot noticed that he was the source of gossip, resulting in the CO’s decision to hold a safety standdown (A safety standdown is a RNLAF intervention that cancels all flights and brings the aviation community of a squadron together to discuss the safety matters at stake). During this safety standdown, the pilot expressed remorse about betraying the trust of his colleagues and made a public promise to do all he could to keep everyone on board safe. After his story, the responses from his colleagues showed understanding, compassion, and acknowledgement [40].

Case 2: Fatigue: During tactical training missions, the entire unit slept in tents, mimicking a situation close to the front of a conflict zone, resulting in an increasingly fatigued flight crew due to sleep disturbances. Although having been addressing this problem for five years, flight crews felt they had not been taken seriously, and they had recently been threatened with personal consequences for their careers if they did not drop the matter. The flight crews had asked for fatigue management rules dictating the sleeping arrangements and both quantity and quality of sleep. Although fatigue management rules were not provided, measures were taken for some of the future training missions [53].

Case 3: WhatsApp: A few years ago, a group of military students expressed their frustration with classes, exams, grades, and teachers in a class WhatsApp group, adding humor to these situations by using pictures and photos. At one point, some military students inserted photos of themselves and teachers into World War II imagery using photoshop. The images were eventually shared outside the WhatsApp group with the defense organization as part of a standalone legal process. The military students were threatened with immediate dismissal and a thorough investigation was conducted. Although the military students were not fired, they did face disciplinary consequences [54].

The four unpublished operational cases were as follows:

Case 1: Surgical: An infant, born with multiple congenital conditions, was dependent on constant medical care and regular surgery. The infant’s life expectancy was low. Complications arose after one particular surgery and were the reason for additional surgical intervention. The infant died shortly after this subsequent surgery. The parents were dissatisfied with the disclosure and explanations the hospital gave them, and they threatened to sue. The hospital then offered a conversation with a retired surgeon, who had no connection to or interest in the clinical process, and who was not involved with this hospital. This surgeon was in a position to address the parents’ questions and concerns. As a result of this conversation (even though the actual medical team was not involved, which is usual for restorative processes or alternative dispute resolution), the family was able to accept the unexpected sudden death of their child as a plausible outcome of the medical care given and was able to start the process of coming to terms with the loss [55].

Case 2: Dangerous goods: A worker specialized in transporting dangerous goods by air was ordered by his supervisor to send a load that was not properly packed nor labelled. Although this non-compliance was pointed out to the supervisor six times by the worker, the load was transported anyway. When the package arrived at the destination, the relevant authority was—by sheer coincidence—conducting an inspection. The findings regarding this particular load resulted in fifteen fines for the worker, each fine worth up to EUR 1500. The fines were rescinded later, but the situation was upsetting and frightening for this worker, who had tried everything possible within their employment relationship to ‘say stop’ [56].

Case 3: Air traffic management: An incident occurred in which the primary voice communication system used by air traffic controllers was becoming increasingly unreliable: instances started occurring where the system would fail completely, with aircraft flying around, unable to contact the controllers. Because of the technical issues, flight traffic was reduced but not ceased—which is what the controllers had requested. They were left feeling pressured to perform their job with an ill-working system and feared an accident could occur for which they would feel (and probably be made) responsible. Although they voiced their concerns frequently and every technical issue was duly reported, they felt unheard, their opinions, experience, and expertise not taken seriously by the organization. They felt trapped in their situation; even after the technical issues were solved, the uncertainty and fear as well as resentment towards senior management persisted [57].

Case 4: Flight crew replay: Flight operations sometimes put crews before unusual situations to which they need to adapt (against the background of goal interactions, time pressures, and limited resources), and not always with adequate procedural guidance from their organization [58]. Surprises or minor operational mysteries in such cases are not uncommon [59], leaving the parties involved with unanswered questions. A major European airline developed a method to bring multiple data traces and human perspectives together by visualizing (or replaying) relevant portions of the flight and second-to-second aircraft systems status. For this to be successful, parties have understood that several preconditions need to be met to ensure full disclosure during the replay. The critical precondition is the a-priori agreement that no other safety or disciplinary investigation will be started once a crew has requested or been invited for a replay [60].

## 3. Findings

Our findings elicited eight enabling conditions whose absence or presence either hampered or fostered a restorative response. They seemed to coagulate around leadership qualities, media and judicial attention, engagement of the ‘second victim’, and leadership apprehension or unease linked to their political room for maneuver in the wake of an incident.

Table 1 shows a few examples of the first and second analysis, resulting in eight enabling conditions (themes) that were reduced to the final three enabling conditions for successful implementation of a restorative just culture. We present the eight in more detail below.

### 3.1. Leadership Response

Leadership has the power and ability to choose how to respond to an occurrence. With this choice, leadership has determined the message it wants to send to its employees, upper management and/or executive board, and other stakeholders. As the studied cases show, it is not always a deliberate decision to respond retributively. Often, this is the result or outcome of a number of more or less unconsciously made decisions. For instance, the shock that leaders experience following an occurrence can result in an emotional bias. As found in the WhatsApp case, leadership, horrified by the images, immediately labeled the involved students as right-wing extremists. This was, however, not the case. Leadership was shown the end result of a process that went on for months and in which the images worsened gradually. Furthermore, the content of the images was related to the specific subject presented in class, which happened to be World War II, and not to the political affiliation of the students. Not being aware of the gradual process and context in which these images were shared, leadership responded, understandably, based on their own moral conviction instead of understanding the context surrounding the behavior and judged this behavior as both immoral and right-wing extremist [54,61,62,63].

### 3.2. Leadership Expectations

On other occasions, the decision to act retributively is deliberately taken because it seems this is the behavior expected in the organization that the leadership is part of. Specifically in hierarchical organizations, the quality of leadership is determined by competences such as decisiveness and task orientation [64]. Dealing with occurrences in a decisive manner therefore has a positive impact on one’s own career. This decisive response is more often than not translated into showing all parties involved and the rest of the organization that the behavior is unacceptable and is met with consequences. This means leadership experiences a pressure to punish [40]. At the same time, a restorative response can be quite difficult as it seems to go against what is expected: a firm response showing strong leadership. Having an example of the benefits of a restorative response can counter this unease. The safety standdown case [40] shows the power of a restorative response, as trust and relationships were restored, and the involved operator often uses this example to teach others.

### 3.3. Perspective of Leadership

What all retributive cases reviewed have in common is that leadership often looks at an occurrence from only one perspective: their own. However, there are different perspectives regarding an occurrence. Realizing that these different perspectives exist opens up the possibility of a restorative response [65,66]. In the surgical case analyzed, the perspective of the parents was positioned next to the medical perspective provided by the retired physician based on the medical records available. What became clear was that for the parents, living with the medical situation of their child for years, surgery was just another aspect of their normal daily routine as they had gotten ‘used’ to the situation over time. The medical perspective, provided in plain language, showed the parents how exceptional the medical conditions of their child were and that complete recuperation was never possible. It was known that new medical complications would arise over time with the child getting older and growing. With both the family and medical perspective of the life of the child, the family started to realize that their child lived a very special and fragile life that was not expected to last long. With the gained insights of both perspectives, the family started to accept that the unexpected sudden death of the child was a realistic possibility as an outcome of the medical care given. After the conversation, the family was able to accept the tragic outcome and start with the process of grieving [55].

### 3.4. Tough on Content, Soft on Relationships

A number of cases reveal an unease felt when speaking up about a situation or the ‘right course of action’. In an earlier published case regarding fatigue among military pilots [53], it became clear that speaking up about the effects of sleeping arrangements on flight safety had a negative impact on how the operators reporting this matter were treated. The fact that management did not agree on the substance of the fatigue issue affected the relationship it had with these employees. As a result of this deteriorating relationship, these employees became more and more reluctant to come forward with safety-related information. They felt they were punished for their outcry. The same effect was found in the safety standdown case [40], in which an operator publicly spoke about an occurrence he had been involved in and was subsequently met with disapproval and disdain from his colleagues.

### 3.5. Political, Public, and Media Attention

Another finding distilled from the cases analyzed pertains to the fear and unease experienced after an occurrence, especially by leaders and upper management. If an occurrence goes public and a (negative) response from politicians and/or the media is to be expected, decisions on how to deal with the occurrence seem to be driven by that prospect [67,68]. Leadership is often pretty good at predicting a possible negative response from the public, press, and/or politicians. For instance, the analysis of the WhatsApp case shows a fear of political backlash if the case was not handled decisively, visible in the statement made public by the Ministry of Defense, in which the measures to be taken against the students and a member of leadership were announced [54]. However, in the newspapers, a news item appeared entitled, “How investigation into Nazi statements within the army was sabotaged,” reporting that the defense organization is worried about its appeal to right-wing radicals and including the quote, “Those statements back then were really racist, there’s no doubt about that.” This media attention led to questions in Parliament.

### 3.6. Regulatory or Judicial Attention to the Incident

In many instances, an incident is accompanied by the (fear of the) possibility of a regulatory or judicial response, be it from outside an organization, i.e., public prosecution, or inside an organization or sector, i.e., being disciplined internally or facing professional disciplinary law. The fear of being prosecuted or disciplined results in less openness about an incident [14,69,70,71] and naturally hampers the possibility of restorative justice. The possibility of public prosecution is something that in most cases falls outside the scope of influence of an organization. However, judicial attention to an incident within an organization is not. The organization itself can still choose how to respond. As the surgical case [55] shows, understanding and openness to the questions and concerns of those impacted by the incident enabled eventual restoration: the family initially rejected explanations provided by the hospital, and their desire for more and foremost better answers to their questions created a conflict between the family and the medical team, resulting in the threat of suing. Instead of countering this threat by following formal complaint procedures or consulting the hospital lawyers, the hospital responded by offering an explanation via a third, unbiased party, regardless of the time (and therefore costs) needed. The tension between the family and the hospital was alleviated, and restoration became possible.

### 3.7. Second-Victim Acknowledgement

An important aspect of the restorative response is being aware of the suffering and needs of the people directly involved in the occurrence. These people are called second victims: practitioners involved in an incident that has an impact on someone else and for which they feel personally responsible [22]. As the safety standdown case shows, involving these second victims is key if recovery, learning, and restoring trust are the goals to be achieved [40]. During this safety standdown, the second victim, up to that point harshly judged by his peers, was provided the opportunity to share his perspective on the occurrence. Although originally intended for the second victim to provide information about the reasons behind his behavior that led to the occurrence, he took this opportunity to express remorse, making an honest apology to his colleagues for betraying their trust and accepting accountability for the situation. Although it was not specifically asked for, the second victim was forgiven and trust among the entire group of peers was restored [40]. The acknowledgement of the second victim resulted in this unit being able to compare perspectives, learn from what happened, and come to an understanding regarding future operations. Quite the opposite happened in the dangerous goods case, where the second victim was not acknowledged. In this case, the worker was not given the opportunity to share his side of the story when working with the load to be transported or the impact of the initial punishment (15 fines). The resulting fear and feelings of betrayal led to the worker experiencing a burn out, needing psychiatric help, and leaving his profession. In 2023, he left the organization, mourning the loss of team spirit he was so proud of before the dangerous goods case happened [56].

### 3.8. Safe Possibility of Full Disclosure

Those who are involved in an undesired event have valuable knowledge about what happened. For others and organizations to understand the course of the event, it is important that those involved are willing to disclose their narrative. Disclosure from the perspective of a restorative just culture is the ethical obligation of those involved with an adverse event to give account of that event, based on a fiduciary relationship: “Disclosure can be seen as a marker of professionalism” [26]. Disclosing one’s perspective openly, after a breach in trust, is not a given. The uncertainty of what consequences a disclosure might have for the involved can hamper full disclosure [26]. To reduce the uncertainty and manage expectations, a reference framework is a necessity.

The flight crew replay case shows that an organization must acknowledge the expertise and experience of its employees as well as show a desire to attain full disclosure. The perspectives of professionals are gathered and used to make sense of (quantitative) data and events. Notwithstanding the preconditions met to ensure full disclosure, the message sent to employees that their insights are valued becomes a precondition in itself. Being seen as a professional with valuable knowledge and contributions creates a climate in which these same professionals are willing to share information and diminishes the chance that the message is met with a retributive response.

Receiving acknowledgement as a professional alone, however, was insufficient for a full disclosure. For flight replays, these aspects helped enable open disclosure:

- A written and mutually acknowledged agreement between the employer and those involved (or representatives, e.g., a union) about the replay process and its rules.
- The session is organized and hosted by a representative of an independent department within the organization: for example, the Safety Department.
- Participants are the crew, the host, a representative of the operator, and a referee, who monitors the replay being executed according to the written process.
- The crew involved with the event can ask others (except the host, who is almost always a crew member too) to leave the room if they want to disclose sensitive information.
- Information that is disclosed during the session will not be used in any written report, nor will it be traceable to the specific crew.
- When a crew participates in a replay session, it will be impossible to start any disciplinary process against the crew afterwards, nor a safety investigation [60].

## 4. Discussion

The analysis of the published and unpublished cases elicited eight aspects that provide insights as to what preconditions are needed to achieve restorative success. These eight aspects are: (1) leadership response, often determined by a number of more or less unconsciously made decisions based on, for instance, the shock that leaders experience following an occurrence; (2) leadership expectations, involving the decisiveness and task orientation expected of leadership in the organization, often resulting in a pressure to punish; (3) perspective of leadership, often the only perspective taken into account, ignoring the other perspectives involved; (4) ‘tough on content, soft on relationships’, in which speaking up about something results in deteriorating relationships; (5) public and media attention, which is the fear and unease about (the possibility of) an occurrence going public and attracting unwanted attention from politicians, the public, and the media; (6) regulatory or judicial attention to the incident, which is the (fear of the) possibility of a regulatory or judicial response from in and/or outside an organization, like disciplinary law or public prosecution; (7) second-victim acknowledgement, which involves being aware of the suffering and needs of the people involved in an occurrence that has an impact on someone else and for which they feel personally responsible; and (8) possible full-disclosure setting, focusing on freely sharing relevant information as well as accountability of all involved. Based on these aspects, we provide an in-depth discussion of preconditions for restorative success in this chapter.

### 4.1. Leadership Qualities

Considering aspects such as leadership response, leadership expectations, the perspective of leadership, and the ‘tough on content, soft on relationships’ attitude, it becomes clear that responding to an incident can be quite a challenge for leadership. The pressures on leadership to act visibly and decisively following an incident can be considerable. For instance, there is the immediate demand for damage control, for mitigation, and for preventing even worse harm from occurring. An event with a bad outcome is likely an economic issue. There can be immediate consequences of a disruption or delay in operations. If the incident is bad enough, work grinds to a halt on that ward or in that operating theater. The loss of an asset or personnel can occur. A regulator could even step in and stop operations, or the incident could attract the attention of other unwelcome sources. This means the event can be a reputational issue too, and even one of job security for the leadership.

This makes it attractive to single out and blame the individual(s) involved, focusing on what they have allegedly done wrong and what they deserve [22]. Leaders can even be seduced by the notion that acting in such a way can be sold in the name of ‘safety’ by fixing or removing ‘bad apples’ from the organization [72]. If they eschew retributive decisiveness, leaders might opt for generic but superficial expressions of compassion (“our thoughts are with…”), hedging about causes (“we will fully investigate…”), or stalling on responsibility (“we will fully cooperate…”). A full treatment is outside the scope of this paper, but it is safe to say that constructive alternatives to the ‘bad apple theory’ have long been in circulation in healthcare, at least in theory [8,73,74,75,76], and occasionally in scripts and guidelines [77].

What do the practical attempts to implement restorative justice show? Beyond leadership knowledge of the restorative alternative to blame and retribution, a critical enabling condition is mustering the courage to act restoratively, i.e., moral courage [40]. Courage comes from vulnerability, of course: in this case, leadership vulnerability to derision, impatience, stakeholder expectations, and even active opposition. To initiate and sustain a restorative pathway, leaders need the integrity to remain committed to upholding their values and principles and act consistently and authentically with them. Of course, when confronted with obstacles and setbacks, leaders require persistence and perseverance to continue working towards their goals, as well as the ability to inspire and mobilize others for their cause—through words, actions, and personal sacrifices and examples [66,78,79].

It also requires a foundational empathy and compassion on part of the leadership: a willingness to see and feel the perspective of the second victim and to possess a genuine concern for justice and human dignity. Humanizing the people involved in the incident is necessary to recognize their impacts and needs and to begin to understand the obligations this puts on leadership. Humanizing the people involved in the incident hinges on actually getting to know the persons—the humans—behind the role or stereotype [80,81], which takes time and effort. It may also create leadership discomfort because of the realization that whereas the care may have flowed through the hands of the people who are now second victims, the harm inflicted was produced systemically, by the organization they as leaders are responsible for [75]. The people involved in the incident did not make bad choices; they had bad choices.

### 4.2. Fear and Unease and the Political Room for Maneuver

A restorative process can initially be met with apprehension and unease. A retributive response is a clear and decisive response, after all, which shows that decision makers ‘take safety seriously.’ A restorative response takes time and might lack any form of classical punishment, thus being seen by some as not morally serious. With politicians, the public, and the media watching, this can feel rather uncomfortable. An organization, especially a public sector one, can be or at least feel to be rather dependent on the image both politicians and the media have of this organization [36,82]. Management of public sector organizations need solid public relations because they need to ensure access to resources and mandates. Management of private sector organizations, on the other hand, need solid public relations to ensure customers and growth potential. The fear of a negative image as a result of an incident can be compounded by management’s perception of personal vulnerability [83]. A restorative process that requires full disclosure in order to restore trust, ensure learning, and move forwards could shed unwelcome light on the role of management in the incident. A restorative process, while repairing the harm done in an incident, might cause additional damage (a deteriorated image of management) for which, management fears, there is no simple solution. An image of being unreliable or unprofessional might be unrepairable and have severe consequences, as can be seen in the resignation of a Dutch Minister of Defence and Chief of Staff in the wake of a severe accident [84,85]. Avoiding the apprehension and unease of unwanted attention from politicians and the public—a bad review, so to speak—becomes an interest of its own [86].

Dealing with these kinds of apprehension and unease is rather complex and may be solvable only up to a certain point. However, the data suggest some things that can be done to heighten the chance of restorative success. The first is to realize that in some instances the restorative process results in a situation that requires another restorative process. If, as the result of full disclosure, a relationship between people deteriorates—for instance, because one party learns about something the other party has done and considers this below the professional standard—then that aspect also requires a restorative process. Ideally, this continues until all the damage, primary and secondary, has been addressed and resolved.

When it comes to fear of reputational damage, research shows that in some instances the possible consequences of an incident are overestimated and this fear is actually unjustified [86]. One way to deal with this fear is by simultaneously testing the likelihood of and responding to possible negative consequences, which can be achieved using a tool created for crisis communication called the Wet van Pleuris (Law of Misery) [87]. According to this law, it is possible to determine how an incident will be perceived by the public and media and what is needed to effectively deal with this perception. Three variables determine how much turmoil might occur: culpability, relevance, and mediagenicity. For each variable, this tool provides several questions to determine the extent to which it exists. These questions can also be used to create a strong narrative when communicating about the incident.

The same can be said of the apprehension or actual possibility of a regulatory or judicial response, such as the application of disciplinary law or the launching of a public prosecution [88]. In the latter case, an organization has no influence on the outcome, but it can control and decide on what response to take to the incident internally, which doesn’t have to be retributive—independent of what happens in the world around. Even if public prosecution becomes a reality, an organization can still respond restoratively. This means that two different processes—one retributive, one restorative—can unfurl roughly simultaneously, where the latter almost invariably helps practitioners (second victims) cope with the stresses and uncertainties of the former [22].

### 4.3. Second-Victim Moral Engagement

The aspects ‘tough on content, soft on relationships’ attitude, second-victim acknowledgement, and a possible full-disclosure setting show that the relationship between the organization/leadership and the employees directly involved in the occurrence of the incident is a critical one.

One of the failure modes of restorative justice is unwillingness on part of the ‘offender’ (a poor choice of words, for sure) or ‘second victim’ to participate in the process [89]. Restorative justice, after all, is relational and aims to repair harm and restore relationships of trust. This is, almost per definition, impossible when key stakeholders are not part of the process. Of course, second victims can have good reasons not to want to participate, and these often go back to a lack of trust in either the process itself or the people who run it or are involved in it [90]. These issues themselves then must become objects for restorative responses, but that obviously requires more work and time, as well as faith in the eventual outcome [2]. Restorative justice is necessarily inclusive and collaborative.

Moral engagement of the second victim is an important enabling condition, and not just to ensure their meaningful involvement in the process. It involves a couple of things (see [91]). First, the second victim must be helped in accepting appropriate responsibility for what has happened. This doesn’t mean that a practitioner should shoulder the blame—quite the opposite. In many cases, accepting appropriate responsibility means that many others in the organization need to engage with their roles and responsibilities. They and their decisions (about equipment, procurement, deadlines, production pressures) may well have helped set the practitioner up for failure. Moral engagement is about embracing the consequences of decisions made in those roles across the organization and finding a balance of who contributed what in steering things toward a bad outcome. It also involves recognizing and acknowledging the seriousness of the harmful effects on others. It is possible to work in an organization, even for a lifetime, and not understand the effects that incidents and their consequences have for those who were affected by them [36,51]. Restoration ideally avoids such dissociation by bringing relevant people together to discuss the impact the incident has had on them and others, achieving full disclosure, and by finding ways to address the harms that came from it together.

## 5. Conclusions

Although a lot has been written about the theory of restorative justice or a restorative just culture, actually achieving restoration in mostly retributive working environments can be quite difficult. Conditions for the ‘fair’ application of retributive mechanisms have long been established, which cannot be said of the conditions for the fair and successful application of restorative practices. In this article, we have explored possible commonalities in the conditions for success across multiple cases and industries. These commonalities can be considered enabling conditions or constraints and can form a frame within which the application of restorative justice can have a sustainable effect. We found eight enabling conditions—leadership response, leadership expectations, perspective of leadership, ‘tough on content, soft on relationships’, public and media attention, regulatory or judicial attention to the incident, second-victim acknowledgement and possible full-disclosure setting—whose absence or presence either hampered or fostered a restorative response. They seemed to coagulate around leadership qualities; media and judicial attention, resulting in leadership apprehension or unease linked to their political room for maneuver in the wake of an incident; and the engagement of the ‘second victim’.

What is needed next is a systematic review of a larger number of existing cases to see if these three enabling conditions—leadership qualities, fear and unease about the political room for maneuver, and second-victim moral engagement—can be found consistently. Furthermore, future research could focus on experimenting with these three enabling conditions in practice in order to achieve a restorative response that is sustainable. In future research, we will therefore explore, and possibly test, if these enabling conditions can be deliberately used to ensure sustainable restorative success.

## Figures and Tables

**Table 1:** The process of analysis, resulting in 8 themes and 3 enabling conditions.

Example Findings (First Round of Analysis)	Cases	Themes (Second Round of Analysis)	Enabling Conditions (Final Round of Analysis)
Upper management experienced severe shock as a result of the pictures. Despite the fact that the content of the WhatsApp group for the cadets was about the humorous element and dealing with boredom and frustration, from the organization, the behavior is labelled as right-wing extremist, racist, transgressive, hurtful, insulting and referring to Nazi Germany.	WhatsApp	Leadership response	Leadership qualities
Despite the amount of pressure, there was very little support from management to help resolve the situation. Rarely did they feel there was support, which made them feel very alone. Although the problem had escalated towards management, nothing was done.	Air traffic management		
Responding in a soft, restorative way poses the risk of derailing one’s career. If COs want to impress their superiors by showing how they personally managed tricky situations, retribution looks better. At the same time, COs feel that when something negative happens, they can no longer be the leaders they aspire to be. Decisions on what should be done are made higher up the chain of command and the COs are tasked with executing the wishes of their superiors without having any say in the matter.	Safety standdown	Leadership expectations	
A good example of the lack of psychological safety in a retributive just culture is the tactical training exercise that in the end permitted operators to sleep in buildings instead of tents. According to the operators, this was possible because the exercise leader had no intention of pursuing a career in the RNLAF and did not care how this decision would affect his image within his chain of command	Fatigue		
The parents and family had many open questions which the medical team tried to answer them from their perspective, medical knowledge and available information. The family, however, did not accept the explanation given and desired more and foremost better answers to their questions. […] The retired doctor gave the family time and opportunity to give their perspective of their child and how they perceived the medical situation from birth till death.	Surgical	Perspective of leadership	
The exact cause, context and intention of the WhatsApp photos has not yet been investigated. However, it has been decided in advance that dismissal of those involved, which are also unknown at the time, is appropriate, despite the warnings not to look solely at political and publicity interests as this does not do justice to the situation.	WhatsApp		
After five years of trying, the operators gave up on the matter, no longer spoke up about the risks they encountered because of the belittling comments and their fear.	Fatigue	‘Tough on content, soft on relationships’	
I did a PowerPoint presentation with a timeline to show exactly what happened, thinking it would lead to a better understanding of my situation. But I had to face some upsetting comments, which made me feel cornered.	Safety standdown		
Communication from defense in the media and towards the Lower House shows that the first focus of top management is on taking action. The interview data show that in the perception of the interviewees, the focus is on coming across decisively to politicians and the media, a clear message that the Ministry of Defense considers such behavior unacceptable and will act harshly against it.	WhatsApp	Political, public, and media attention	Fear and unease and the political room for maneuver
Deciding ‘I won’t do it’ is a very difficult decision when there is so much pressure regarding the progress of military operations at the airports and commercial air traffic at Eindhoven. In theory, you may and can say ‘stop’, but in practice you hardly do so.	Air traffic management		
The situation evolved into a conflict between the family and medical team, ending with the threat of juridical measures initiated by the family.	Surgical	Regulatory or judicial attention to the incident	
The observations made by the KMGCS led to 15 citations against the person concerned and each of these citations could mean 1500 euros fine.	Dangerous goods		
See examples above	Fatigue	‘Tough on content, soft on relationships’	Second-victim moral engagement
See examples above	Safety standdown		
Pilot X acknowledged his role in the occurrence and expressed his sincere remorse, both of which made him feel very uncomfortable. […] And when it was time to ask questions, there were hardly any, mostly comments that showed understanding, compassion, and acknowledgement.	Safety standdown	Second-victim acknowledgement	
It was eventually decided to dismiss the case saying that this should never happen again. The person concerned literally never did another transport. He now works in another field. The person concerned never wants to have another conversation with his former manager because there was never any acknowledgement of his situation or repentance/remorse from his manager.	Dangerous goods		
A session where crews are willing to give as much disclosure as possible about their perspective of the situation. […] Once the crew is invited for a Replay, no other safety or disciplinary investigation will be started any more. Even not about what is disclosed during the Replay session.	Flight crew replay	Safe possibility of full disclosure	
Operators felt that from a certain rank up, commanding officers were reluctant to allow crew to speak up about difficult matters. Metaphorically this behavior is called the watermelon syndrome: what is red on the inside—the operator level—becomes green on the outside—the level of airbase commander.	Fatigue		

## Data Availability

To access the full data, a formal request can be made to the Department of Defence of the Netherlands.
